# The Principles of Progress

**Published:** 1888-02-25

**Authors:** 


### The Hospital

A Weekly Institutional Journal of

**Science, Medicine, and Philanthropy.**

*For Hospitals, Asylums, Medical Practitioners, Students, Nurses, Families, Parishes, Congregations, and the Charitable Public.*

Vol. III. No. 74.] [February 25, 1888.

### Principles of Progress.

It is an axiom familiar to all men, that social and political organisations, like living beings, cannot long exist in a stationary condition; they must either advance or recede. "Change" is written upon all things. On some it is "change and growth"; on others, "change and decay." In youth there is a forward outlook, an ambition for progress, a resolution for success. Men and women of strong individuality are restless, and eager to attain some coveted position, some far-off goal of real or fancied good. They spare no strenuousness of thought, leave no effort unmade that may bring them to the eminence which in the distance satisfies the eyes of their desire. This temper of mind, which is the seed-bed of all great deeds, has in many people but a brief duration. They reach some little vantage-ground where continued existence is possible without much exertion of mind or body, and then the indolence which is natural to human creatures asserts itself, and whispers to not unwilling ears, "It is enough; this is a pleasant camping-ground, where storms are not many or great; why not remain here and be content?" The majority of men and institutions, when once such moderate vantage-ground is reached, are content. They make no further special efforts for themselves, and resent all spurrings and stimulations to such effort which come to them from the outside world. Then the law of change begins to operate in a backward direction: they have ceased to desire growth and expansion, and growth and expansion, in answer to their wish, have ceased to belong to them. Decay and disintegration have become their companions instead.

Good institutions, like good men and women, are as essential to the world's well-being as air and sunshine. But a human institution is composed of various parts, like a watch; and in order that it may do its work well, like a *good* watch, it is necessary that each part be perfect of its kind. The better the institution and the higher the purpose it serves, the more necessary is it that as a whole and in each of its parts it should be as perfect, as efficient, and as enduring as it can be made. A Church, for example, which teaches and sets before the people models of duty towards God and men, should, if possible, have teachers whose wisdom is unquestionable, and whose virtue is of the purest kind. When these conditions are fulfilled the Church lives, grows, purifies, and in every way accomplishes the ends for which it has been founded. But when the Church, in its teachers and people, fails largely to be either wise or virtuous—and examples of such failure are not unknown to history—then that which was good becomes evil, that which was wholesome and pure becomes pestilential and destructive, that which deserved and obtained the approval of God and men becomes hateful and intolerable to both.

Will it be contended by any large number of persons that hospitals and other charitable institutions possess no features such as benevolence, purity of motive, and usefulness of service, which give them a character well deserving the name of "sacred"? Is not the hospital, in its place and measure, as essential to the well-being of man and as purifying to the moral and intellectual atmosphere as the Church? And does it not demand for its perfect ideal that it shall be administered in all its departments by men and women of the worthiest personal character and the highest professional efficiency? But a hospital, as a whole, can only be the resultant of all its parts. If its physicians are skilful and kind, but its nurses careless, unsympathetic and inefficient; if its governors are able and diligent, but its official administrators lazy and unconscientious; what can the institution be but an incongruous mixture of good and evil—a disappointment, a disgust, and an injury to all right-minded men?

In the conduct of a purely benevolent organisation, like that of a hospital, the question of "motive" is not secondary, but primary. A merchant or a shopkeeper may have no other motive in the management of his business than that of self-advancement. If hospital officials have no higher inspiration than this, they will never make of their particular institution all that it is capable of being, and all that it ought to be. Doubtless, as a matter of experience, it is found that even the best men will sometimes be influenced by motives which are more or less mixed. But that is no reason why the ideal hospital official should not desire to be inspired by feelings of the purest benevolence, and constantly strive to bring his motives to an ideally perfect standard. The man or the woman who does not believe in purity and perfection of motive is already far on the way to baseness of life. He who professes self-advancement as the first article of his creed, and proclaims his conviction that it is the first and last article of everybody else's creed too, is of the same religion as the animals that make no claim to intellect or morals. As an indispensable condition of the highest progress in hospital life and work, there must be the highest motive of benevolence and devotion to duty on the part of those who undertake every department of service. It is only a good tree which can by any possibility bring forth good fruit.

Efficiency in work is hardly less important than simplicity of intention; many people, indeed, think it more important. Its relative position as a factor of progress need not be discussed. Of its absolute value there can be no doubt. Perfect efficiency is only possible to high motive. That degree of attainment is so rare as to be almost unknown; and yet if perfection is never aimed at, even moderate success will hardly be reached. In the case of a doctor or a nurse the incentives to excellence are so many that it is difficult to believe that any hospital physician or nurse can ever be content with a perfunctory and second-rate performance of duty. The sick man or child is so helpless, so absolutely at the mercy of his attendants, so trustful of their skill and kindness, that the man or woman who would be lazy or less than kind at the bedside would deserve to be turned out of the ward in disgrace.

Last mentioned, but inferior to no other essential condition of the highest hospital management, is that of personal loyalty. Those whose work lies within the walls of a hospital must be deeply and sincerely loyal to their own ideal of duty. No want of such devotion on the part of others should ever tempt them to a lower standard of service. True loyalty demands the ungrudging recognition of the just authority of governors and managers, and a willing and hearty obedience to their directions. Hospital committees, like other bodies of men, may make mistakes, and such mistakes may need to be pointed out and corrected; but the committee is the properly-constituted authority, and there cannot be the least doubt that as a rule the desire of its members is to promote in every way the prosperity and efficiency of the hospital. In this desire they have the fullest right to expect and to receive the heartiest and most thorough co-operation of every official in the hospital —from the senior physician and matron down to the newest probationer nurse and the house-surgeon who was appointed but yesterday. A like honest loyalty should be accorded by subordinates to official superiors, by superiors to subordinates, and by equals one to another. It is not the mark of a free and generous mind to be always suspecting or rebelling against authority; rather is it an evidence of vulgarity and want of intelligence. He who sees clearly and deeply into life knows that order and good work are only possible when every man does his duty well and thoroughly in his own place. He knows that the subordinate of to-day may be the superior of to-morrow, and that no man can rule well who has not first learned to obey. Schism is a serious, and may be a fatal, mistake, and can only be justified when no other course is possible to an honest and candid mind. We have ventured to put these considerations before our readers, feeling that in the stress and storm of life great principles are liable to be forgotten, and that when they are forgotten the perfunctory and comparatively valueless service of a slave takes the place of the prompt and spontaneous duty of a free man. Hospitals can only truly fulfil all their high and benevolent functions when each worker in his own sphere resolves and strives to make his own work complete and perfect.

